# Chemically Polymerized Polypyrrole on Glucose-Porcine Skin Gelatin Nanofiber as Multifunctional Electrochemical Actuator-Sensor-Capacitor

**DOI:** 10.3390/polym17050631

**Published:** 2025-02-26

**Authors:** Rudolf Kiefer, Toribio F. Otero, Madis Harjo, Quoc Bao Le

**Affiliations:** 1 Conducting Polymers in Composites and Applications Research Group, Faculty of Applied Sciences, Ton Duc Thang University, Ho Chi Minh City 700000, Vietnam; lequocbao@tdtu.edu.vn; 2 Centre for Electrochemistry and Intelligent Materials (CEMI), Universidad Politécnica de Cartagena, Aulario II, Paseo Alfonso XIII, E-30203 Cartagena, Murcia, Spain; toribio.fotero@upct.es; 3 Intelligent Materials and Systems Lab, Institute of Technology, University of Tartu, Nooruse 1, 50411 Tartu, Estonia; madis.harjo@gmail.com; 4 National Institute for Materials Advancement, Pittsburg State University, Pittsburg, KS 66762, USA

**Keywords:** NFs-PPy, ECMD measurements, three electrolytes in PC, sensor-actuator, energy storage

## Abstract

Multifunctional materials requiring low functional voltages are the main goal of new industrial smart technologies. Polypyrrole (PPy) was chemically synthesized by a simple dip-coating process on glucose–porcine skin gelatin nanofibers, accelerating mass production, here shown on nanofiber scaffolds (NFs) with those consisting of composites. The isometric and isotonic characterizations by electro-chemo-mechanical deformation (ECMD) of NFS-PPy are obtained from cyclic voltammetric and chronoamperometric responses in lithium bis(trifluoromethanesulfonyl)imide (LiTFSI), lithium triflouromethanesulfonate (LiTF) and sodium perchlorate (NaClO_4_) in propylene carbonate (PC). The results indicate a prevalent anion-driven actuation of the linear actuator (expansion by oxidation and contraction by reduction). Different stress (4–2 kPa) and strain (0.7–0.4%) gradients are a function of the anion Van der Waals volume. During reversible actuation (expansion/contraction), the material stores/releases energy, obtaining greater specific capacitance, 68 F g^−1^, in LiTFSI solutions, keeping 82% of this capacity after 2000 cycles. The sensitivity (the slope of the linear sensing equation) is a characteristic of the exchanged anion. The reaction of the PPy-coated nanofiber is multifunctional, developing simultaneous actuation, sensing, and energy storage. The materials were characterized by scanning electron microscopy (SEM), energy dispersive X-ray (EDX) spectroscopy, and Fourier transform infrared (FTIR) spectroscopy.

## 1. Introduction

The industrial demand for sustainable materials with multifunctional biomimetic responses that have a long active life, adapt to mass production, and consume low functional energy is increasing, but those multifunctional materials are challenging. Conducting polymer composites with electrochemical multifunctional and biomimetic responses are being explored as candidates. These conducting polymers are applied in electrolytes (liquid or solid), and respond at low voltages, giving simultaneous actuating [1,2], sensing [3,4], and energy storage [5] functionalities. Most conductive polymers, such as PPy, are electrochemically polymerized to achieve “artificial muscle” adaptation [6] of high stress [7], strain [8], and strain rates [9]. The main drawbacks that used to be considered for the industrial application of electrochemically polymerized PPy are the electrochemical protocols during both the polymerization and the actuation processes. Changing polymerization techniques [10], current densities or potentials [11], temperature [12], and other parameters can improve the actuation properties of the attained PPy. Alternatively, PPy can be obtained by chemical oxidation of the monomer [13] for actuators or coating nanowires [14], for energy storage and sensors, or as anti-oxidants [15] or anti-corrosion [16] films. The chemical deposition of PPy on fibers, textiles, or other substrates enables mass production due to the simple dip-coating process. Both chemically and electrochemically generated PPy materials are faradaic actuators.

The charge density consumed during oxidation/reduction drives the exchange of counterion and solvent with the electrolyte for charge balance in the PPy film and osmotic balance with the electrolyte [17]. Consequently, the PPy material expands/contracts, originating linear or bending reversible actuation driven by the reaction-induced volume changes. The electrochemically stimulated conformational relaxation (ESCR) model provides a good quantitative description of the actuation [18]. The energy consumed by the actuating reaction, or any components (reaction current or reaction potential), responds to and senses the working physical and chemical conditions. The device is multifunctional: one actuator and several sensors work simultaneously, driven by the same reaction [19]. The actuating (electrical charge) and the sensing (potential evolution or current evolution) signals are present in the two connecting wires at any actuation time. In addition to using conducting polymers as electrochemical sensors, other composites, such as titanium dioxide nanoparticles/multi-walled carbon paste electrodes [20,21], have demonstrated selective responses to larger molecules, including the rafoxanide drug substance. Generally, the mechanism of MWCNTs follows a non-faradaic process involving ion injection, where the material’s pore size plays a critical role.

In this work, we will explore the multifunctional properties of PPy chemically oxidized on nanofiber scaffolds (NFs-PPy), allowing simultaneous sensing actuators to store and release electrical energy during actuation. In order to advance the influence of the electrolyte over that multifunctionality, three different salts dissolved in PC will be explored: LiTFSI, LiTF, and NaClO_4_. So far, to the best of our knowledge, the multifunctional approach in organic electrolytes using NFs-PPy has not been explored.

For traditional electrochemical sensors, various techniques are being applied [22]. In this study, multifunctional oxidation/reduction reactions will be investigated using cyclic voltammetry and square-wave current methodologies, which require specific conditions for meaningful results. Regardless of the experimental conditions, the oxidation/reduction charge (charging/discharging) of the NFs-PPy material must remain balanced. Charge imbalance may indicate simultaneous irreversible reactions, leading to over-oxidation or over-reduction processes that can distort and falsify the quantification of the three studied functions.

Isotonic and isometric ECMD as linear actuation, stress, and strain variations will be followed by applying consecutive potential cycles (cyclic voltammetry) or consecutive square current waves (chronoamperometry) to the PPy-coated nanofibers in the three selected electrolytes. The specific capacitance stored by the actuator will be analyzed from the chronopotentiometric responses using different current densities and the consumption of the same charge density every time. Additionally, sensor calibrations are conducted to evaluate if these three electrolytes, with regard to the ions, can be distinguished from each other and functionalize as ion sensors. The generated materials were characterized by SEM surface, cross-section microscopy, EDX, FTIR spectroscopy, and conductivity.

## 2. Materials and Methods

### 2.1. Getting NFs and Tissues

**Scheme 1:**
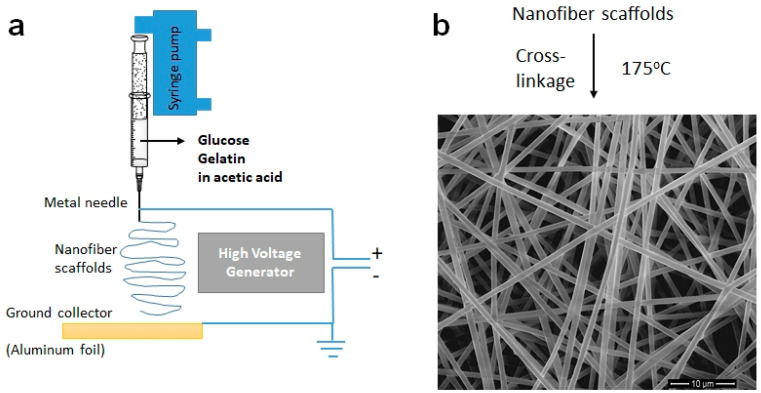
Electrospinning of glucose–gelatin mixture in acetic acid is shown in (**a**). SEM surface image (scale bar 10 μm) of the dry nanofiber scaffold (NF) network is presented in (**b**).

The nanofibers were produced by electrospinning a mixture of gelatin type A from porcine skin and D (+) glucose (99.5%) dissolved in 10 mol/L glacial acetic acid (99%). All chemicals are obtained from Merck (HCMC. Vietnam) and used as supplied. The glucose/gelatin ratio was 1/10, and the mixture was stirred for 2 h. Scheme 1a,b present the electrospinning procedure with the obtained NF scaffolds.

The syringe (5 mL) with the metal needle from Scheme 1a contains the glucose–gelatin solution. The metal needle bottom was located at 14.5 cm from the ground collector (aluminum foil) using a high voltage generator (Heinzinger LNC 3000, Heinzinger Electronics GmbH, Rosenheim, Germany) to apply 17.5 kV. With the help of the syringe pump (New Era Pump Systems NE-511, Farmingdale, NY, USA) in 5–7 μL/min, the glucose gelatin was spun to nanofiber scaffolds (Scheme 1a). The nanofiber scaffold was heated at 175 °C for 3 h to cross-link the nanofiber structure [21]. The NFs’ surface is shown in Scheme 1b, the SEM image of the nanofiber scaffold. The average NFs’ diameter was measured with a micrometer gauge meter (Mitutoyo Digimatic IP65, sensitivity 1 μm, Kanagawa, Japan) was 20.2 ± 1.6 μm.

### 2.2. Coating the NFs with Chemically Oxidized PPy

**Scheme 2:**
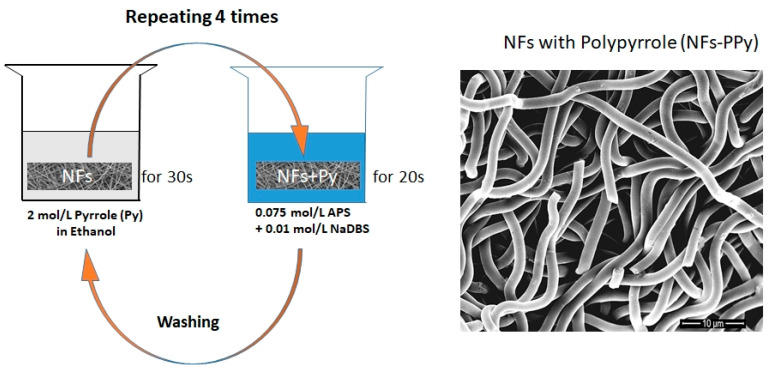
Coating procedure of NFs with PPy using APS with NaDBS as aqueous oxidant solution with additional washing and drying steps. The cycle was repeated 4 times giving the NFs–PPy material presented in the SEM surface image (scale bar 10 μm).

The chemical oxidants obtained from Sigma-Aldrich (Taufkirchen, Germany) were 0.075 M ammonium persulfate (APS, 98%) dissolved in deionized water with 0.01 M sodium dodecylbenzenesulfonate (NaDBS, technical grade) used as electrolyte providing the counterions to the oxidized PPy. The coating procedure is presented in Scheme 2.

The coating procedure requires two steps. First, the NFs were immersed in 2 M pyrrole (Py, 98%, Sigma-Aldrich, Taufkirchen, Germany) solution in ethanol for 30 s. Then, it was transferred for 20 s into the oxidant aqueous solution (APS/NaDBS), with gentle stirring applied. The coated NFs-PPy was then washed several times using deionized water and ethanol in order to remove both oxidant and unreacted pyrrole monomer. The chemicals APS, NaDBS, and ethanol are purchased from Merck and used as supplied. Any PPy weakly bound to the NFs surface was eliminated by subsequent mechanical rubbing. The chemical coating and washing procedure was repeated 4 times in order to guarantee a uniform and thick PPy film coating on each nanofiber. The NFs-PPy was then dried under supercritical CO_2_, to obtain an overall average diameter of NFs-PPy in the range of 32.4 ± 2.2 μm.

### 2.3. ECMD Measurements of NFs-PPy

The NFs-PPy samples were cut into pieces that were 9 mm in length and 1 mm in width. Then the various samples were immersed for 24 h prior to linear measurements in every one of the 0.2 M propylene carbonate (PC) solutions of the three studied electrolytes: lithium bis(trifluoromethanesulfonyl)imide (LiTFSI, 99.95%, Solvionic, Toulouse, France), lithium triflouromethanesulfonate, LiCF_3_SO_3_ (LiTF, 99%, Merck, HCMC, Vietnam) and sodium perchlorate (NaClO_4_, 98%, Merck, HCMC, Vietnam), which were used as supplied. One end of the NFs-PPy samples was fixed in one of the two gold-coated clamps of the static arm from an electrochemical cell, allowing contact to the working electrode exit of the potentiostat. The other end was connected to the force sensor (TRI202PAD, Panlab, Barcelona, Spain) and the home-made linear muscle analyzer setup [22]. The counter-electrode (platinum sheet) and the reference-electrode (Ag/AgCl (3M KCl)) were also connected to the concomitant potentiostat (Biologic PG581, Seyssinet-Pariset, France) exit. The electrochemical signals from potentiostat and signals from the ECMD were combined in a new home-made software [22] to perform measurements in real-time. Either isometric ECMD (constant length between both clamps of 1 mm) to determine change of mass (calculated in stress σ = F/A with F as force and A is the cross-section of the NFs-PPy) or isotonic ECMD (constant mass of 100 mg, force of 1 mN) to determine the length change (calculated in strain Δl/l × 100 (%) with Δl as the fractional length change and l the original length) were applied. The linear muscle analyzer has a movable stage where the k factor (mg/µm) as a calibration tool is determined to analyze the mass-to-length change made before and after measurements.

Before experiments, the NFs-PPy samples were stretched at 0.1% strain for 12 h inside the studied electrolyte. Electrochemical programs such as cyclic voltammetry (scan rate 5 mV s^−1^) and chronoamperometry (frequencies 0.0025–0.1 Hz) combined with isometric and isotonic ECMD were conducted.

The average thickness of the attained NFs-PPy electrolyte samples was 34 ± 2.5 μm, with an average mass of 372 ± 31 μg in the electrolyte. The NFs fiber weight after storage in electrolytes showed the same dimensions (9 mm length and 1 mm width), a weight of 124 ± 9 μg. The weight of deposited PPy on NFs was 248 ± 20 μg. Chronopotentiometric measurements with varied current density i/m (current/mass with mass taken from deposited PPy) of ±0.2 A g^−1^, ±0.4 A g^−1^, ±0.8 A g^−1^, ±2.0 A g^−1^ and ±4 A g^−1^ were applied, having the same charge densities at each applied current density of ±40 C g^−1^.

### 2.4. Microscopic, FTIR, EDX and Conductivity Characterizations

Surface and cross-section (broken in liquid nitrogen) images of NFs and NFs-PPy were obtained from an SEM microscope (Helios NanoLab 600, FEI, USA). FTIR spectroscopic studies (Bruker Alpha with Platinum ATR spectrometer, Billerica, MA, USA) were performed on NFs and NFs-PPy samples between 3500 cm^−1^ and 400 cm^−1^. The EDX analysis (Oxford Instruments with X-max 50 mm^2^ detector, High Wycombe, PA, USA) of the dry NFs-PPy was attained. The NFs-PPy, after polymerization, underwent subsequent washing several times with deionized water and ethanol. After actuation cycles in each different applied electrolyte (LiTFSI, LiTF, and NaClO_4_) the fiber samples were oxidized at 1.0 V for 3 min, then the fiber was extracted from the electrolyte and a small piece was cut, rinsed, dried, and used for the EDX analysis. The remaining sample was returned to the electrolyte, reduced at −0.55 V for 3 min, rinsed, dried, and submitted to EDX analysis. Three different NFs-PPy samples were used in each studied electrolyte for the linear actuation characterization and analysis. The electronic conductivity *σ_e_* of the NFs-PPy samples was obtained with a conductivity meter (LT Lutron, DM-9020, Coopersburg, PA, USA), obtaining the resistivity R of the samples at different locations. The length l between the two contact points (in general, 1 mm) and the cross-section area A of the NFs-PPy samples give the conductivity σ_e_ using Equation (1) [23].

(1) $$\sigma_{e}=\frac{l}{R\cdot A}$$

## 3. Results and Discussions

Only a few studies are using chemically polymerized PPy as an actuator. Most chemically synthesized PPy layers on non-conductive polymers and materials were used as conductive coatings for the subsequent electropolymerization of a PPy deposit [24]. As seen from Scheme 1 and Scheme 2, the formation of chemically deposited PPy revealed linear actuation in organic or aqueous electrolytes [25]. The nanofiber scaffolds mimic muscle fibers and can be applied to artificial muscles [6]. In this work, we will explore and demonstrate the biomimetic simultaneous multifunctionality of NFs-PPy as an actuator–sensor and energy storage device.

### 3.1. Characterization of NFs-PPy

**Figure 1:**
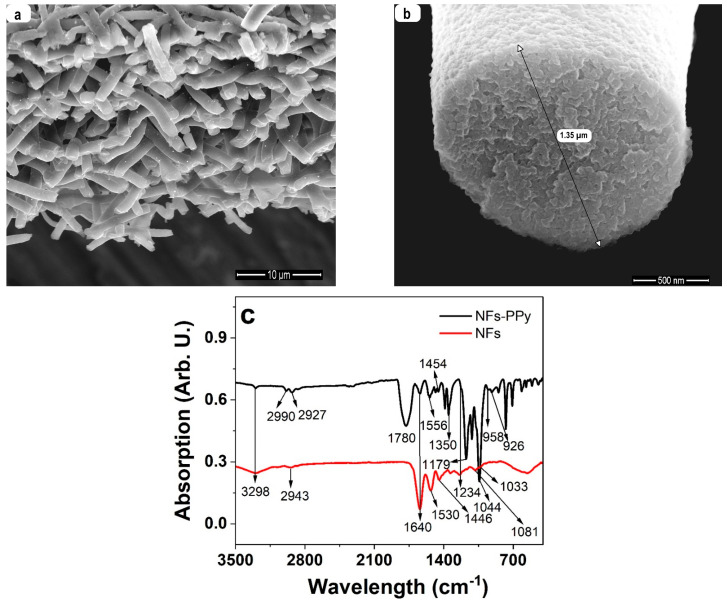
(**a**) SEM cross-section image (scale bar 10 µm) of bulk NFs-PPy, (**b**), single NFs-PPy fiber SEM cross-section image (scale bar 500 nm), and (**c**) FTIR spectroscopy (3500–400 cm^−1^) obtained from NFs (red line) and from NFs-PPy (black line).

Figure 1 presents the NFs-PPy SEM cross-section image of the bulk sample, and Figure 1b shows the cross-section image of a single fiber. The uncoated NFs single fibers are shown in Figure S1. Figure 1c depicts the FTIR spectra attained from NFs and NFs-PPy samples.

The average thickness of the cross-section image of the bulk NFs-PPy sample in Figure 1a is 25.4 µm. The fibers are randomly oriented. Free space is observed between the fibers. The average thickness of the dry bulk NFs-PPy samples after being stored in electrolytes increased up to 34 ± 2.5 µm.

The diameter of the single NFs-PPy fiber, Figure 1b, is 1.35 µm and the average diameter of the NFs fibers (Figure S1) was 0.8 ± 0.06 µm [21] resulting in an average thickness of the PPy deposited on each fiber of 0.55 ± 0.04 µm. The PPy generated on each nanofiber presents a smooth surface, Figure 1b.

The average electronic conductivity of the NFs-PPy sample after formation was 0.36 ± 0.02 S cm^−1^, decreasing after the actuation cycles to 0.28 ± 0.02 S cm^−1^. Previous research using poly(lactic-co-glycolic acid) nanofiber chemically coated with PPy showed three times higher resistivity of 1.7 kΩ/square [26] than our samples with 580 ± 30 Ω/square. PPy-poly(ε-caprolactone) nanofibers were described as having a resistivity of 48 kΩ/square [27]. Chitosan collagen nanofiber scaffolds cross-linked with glutaraldehyde and then coated, using the same oxidant, with PPy [28] had a very low conductivity, in the range of 0.16 S m^−1^. The low resistivity (high conductivity) of our samples can be attributed to the repeated chemical deposition (Scheme 2) process giving a homogeneous and thick PPy coating. The addition of NaDBS to the oxidation solution providing a large counterion to the oxidized polypyrrole during the polymerization–oxidation resulting in a smooth and uniform PPy deposition avoiding the presence of PPy particles overlapping the neighboring NFs.

The FTIR result from Figure 1c depicts characteristic bands of NFs (glucose gelatin) and NFs-PPy with a band at 3298 cm^−1^ related to the hydrogen bonds of the bound water in gelatin [29], shown as well in NFs-PPy. The band at 2943 cm^−1^ of NFs presents the C-H stretching vibrations, while those bands are shown in NFs-PPy at 2990 cm^−1^ and 2927 cm^−1^. The band at 1640 cm^−1^ of NFs is also shown in NFs-PPy related to the amide I group [29]. The 1530 cm^−1^ to the amide group II (N-H bending) are overlapped in NFs-PPy due to the strong vibration of bands nearby. The amide III group-specific band is shown at 1234 cm^−1^, and the small shoulder is in NFs-PPy. Further specific NFs bands are shown at 1446 cm^−1^, which relate to gelatin CH_2_ bending vibration [30], and 1081 cm^−1^ (also shown as a minor shoulder from NFs-PPy) and 1033 cm^−1^ bands belong to glucose (CO vibrations) [31].

In previous research, an increase in the amide bands was found with the shrinkage of glucose bands, verifying the cross-linkage of gelatin assisted by glucose [32]. The NFs-PPy presents a strong band at 1780 cm^−1^ (C=O stretching vibration) related to overoxidized PPy [15]. Other PPy typical bands are present at 1542 cm^−1^ (C=C stretching vibration [33]), 1454 cm^−1^ (C-C stretching vibration [34]), 1350 cm^−1^ (C-C in ring stretch [35]), 1174 cm^−1^ (in-plane C-H vibrations [36]) and 1044 cm^−1^ (=C-H in plane vibration represent aromatic PPy nature) [37]. The oxidized PPy gives bands at 958 cm^−1^ (ring deformation, polaron) and 926 cm^−1^ (bipolaron) [35] with a higher bipolaronic content, confirming the charged (oxidized) state of the just generated NFs-PPy. Figure 1c corroborates the PPy chemical polymerization on the NFs.

**Figure 2:**
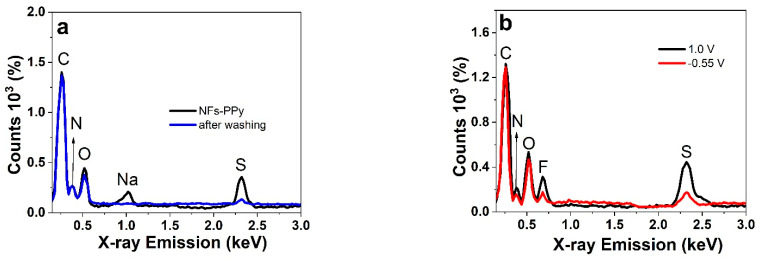

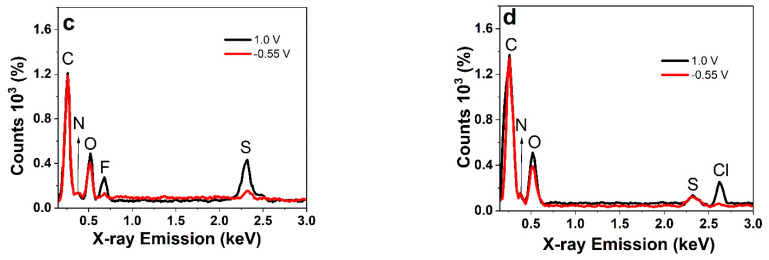
EDX spectroscopy from NFs-PPy samples, (**a**) just after polymerization in oxidized form (black line) and after washing in aqueous solvent and ethanol (blue line); (**b**), after linear actuation in LiTFSI-PC and subsequent polarization for 3 min at 1.0 V (black line) or subsequent reduction at −0.55 V, (red line); (**c**) similar after actuation in LiTF-PC solutions and (**d**), after actuation in NaClO_4_-PC solutions.

Further analysis of the NFs-PPy composition (EDX spectroscopy) is presented in Figure 2. Spectra depicted in Figure 2a were obtained from NFs-PPy just after formation and after subsequent washing. Figure 2b presents similar results after linear actuation in LiTFSI-PC electrolyte and oxidation at 1.0 V or reduction at −0.55 V. Figure 2c presents oxidized and reduced material after actuation in LiTF-PC solutions, and Figure 2d, after actuation in NaClO_4_-PC solutions.

Typical EDX spectra from NFs-PPy just after polymerization are shown in Figure 2a, with element signals of carbon (C) at 0.26 keV, nitrogen (N) at 0.38 keV, oxygen (O) at 0.52 keV and sulfur (S) at 2.32 keV. The oxidized pyrrole (PPy^n+^) generated in aqueous solutions with APS and NaDBS keeps the charge balance with SO_4_^2−^ or HSO_4_^−^ ions (large S signal), as described in previous research [38]. The Na signal (Na) at 1.04 keV indicates that some NaDBS remains trapped in the PPy material. After washing (Figure 2a), the Na signal was removed from most of the sulfur and some of the oxygen signals.

After linear actuation in LiTFSI-PC solutions (Figure 2b), the fluoride (F) signal at 0.68 keV and the sulfur signal are mainly present in the oxidized material, pointing to the exchange of anions during actuation (this equipment does not detect the Li element). The strong diminution of the sulfur signal after reduction is shown from previous research [13], which may indicate the expulsion of the remaining SO_4_^2−^ and HSO_4_^−^ ions with TFSI ions. In summary, the results indicate the entrance of TFSI^−^ anions from the solution driven by the oxidation reaction and their expulsion driven by the film reduction. Similarly, Figure 2c,d indicate the exchange of concomitant anions, TF^−^ and ClO_4_^−^ from the respective solutions. A minor sulfur signal from the reduced samples indicates the minor presence of SO_4_^2−^ and adsorbed DBS^−^ in the PPy film.

### 3.2. Isometric and Isotonic ECMD Measurements

**Figure 3:**
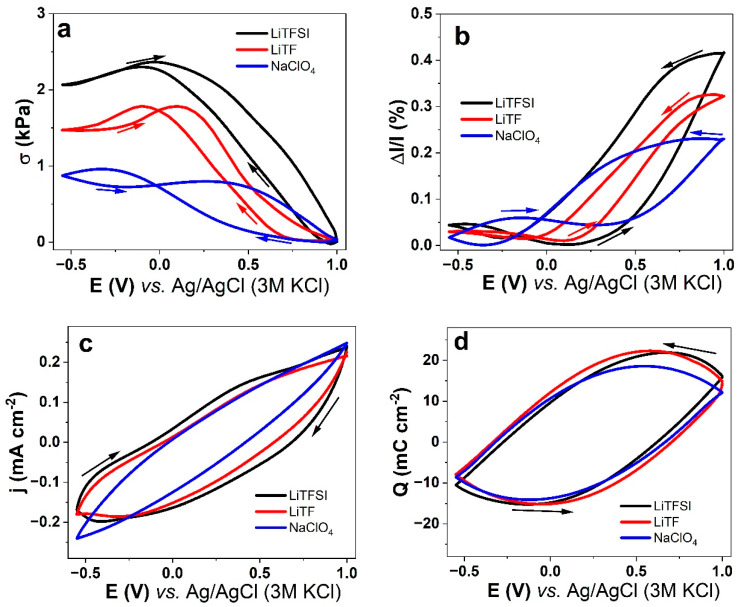
Cyclic voltammetric response (scan rate 5 mV s^−1^, 3rd cycle) of NFs-PPy using three different PC solutions: LiTFSI (black line), LiTF (red line) and NaClO_4_ (blue line) in the potential range 1.0 V to −0.55 V. The isometric ECMD measurements show the stress σ in (**a**), the strain Δl/l in (**b**), the current density j in (**c**) and the charge density Q in (**d**) against the potential E applied to the actuator. The arrows indicate how the magnitudes evolve with the potential scan. The minimum stress is normalized to zero and stress contraction correlates to strain expansion.

Coated PPy nanofibers were reversibly oxidized and reduced in three different electrolytes, LiTFSI, LiTF, and NaClO_4_, in PC solutions by the application of consecutive potential cycles following simultaneous linear actuation. Figure 3a presents the stress evolution (isometric ECMD), and Figure 3b presents the strain evolution (isotonic ECMD). Figure 3c depicts the stationary cyclic voltammetric response (current density evolution), which integration provides the evolution of the consumed charge density (coulovoltammetric response) shown in Figure 3d. The applied potential range was between 1.0 V and −0.55 V at a scan rate of 5 mV s^−1^. These results correspond to the third consecutive potential cycle to obtain a stationary response.

Figure 3a,b indicate a main anion-driven actuation in the potential range between 0.0 and 1.0 V. The PPy-coated nanofiber expands during oxidation with the entrance of counterions and solvent from the solution for charge and osmotic balance and contracts during reduction with the expulsion of counterions and solvent. From 0.0 V to −0.5 V, a minor exchange of cations is present (expansion by reduction and contraction during oxidation). The actuation in LiTFSI solutions presents the largest stress, 2.08 kPa followed by LiTF solutions, 1.47 kPa, and NaClO_4_ with 0.87 kPa. As expected, strain variations from the NFs-PPy actuator present in Figure 3b have a similar tendency of LiTFSI (0.42%) > LiTF (0.32%) > NaClO_4_ (0.23%). The NFs-PPy in the electrolytes LiTFSI and LiTF following a minor reduction expansion of 0.035 % in the strain due to the entrance of cations to compensate for negative charges in DBS^−^, TFSI^−^ or TF^−^ anions trapped in the films. The dominant exchange of anions during the actuator-driven electrochemical reactions (fluoride and sulfur, or chlorine content) was corroborated by the EDX spectroscopic characterization of the oxidized and reduced actuators (Figure 2a,b). Previous studies from NFs-PPy using LiTFSI aqueous solutions also present anion-driven actuation [13].

Chemically formed PPy has been mainly used as a conductive coating and for other applications [39]. Linear actuation studies were performed using a second film of electropolymerized PPy on the chemically generated initial PPy film. The exchange of anions that prevail in electrolytes has been studied. The above-attained actuation seems dependent on its Van der Waals volume. TFSI^−^ (147 Å^3^ [40]) and TF^−^ (80 Å^3^, non-spherical form, and delocalized charge [41]) anions have a very low solvation degree in PC and move as a single entity [42] in PPy. The ClO_4_^−^ anion has a much smaller Van der Waals volume [40] of 54 Å^3^ and low solvation [43] in PC. Therefore, if we neglect the weak solvation in PC, the van der Waals volume of those anions seems to be the main factor for the stress and strain responses of NFs-PPy during the actuation-driven oxidation/reduction reactions.

The stationary voltammetric (current density) responses, Figure 3c, for NFs-PPy in LiTFSI-PC and LiTF-PC have a weak oxidation wave at 0.38 V. The reduction wave for NFs-PPy in LiTFSI is located at −0.41 V, and that from LiTF solutions is at −0.3 V. The NFs-PPy in NaClO_4_-PC electrolytes only show large oxidation and reduction waves, indicating the reaction’s important resistivity. The coulovoltammetric responses (Figure 3d) from NFs-PPy show whatever the applied electrolyte is a closed loop. The polymer oxidation charge equals the PPy reduction charge, indicating the absence of any irreversible reaction in the studied potential range [44], avoiding polymeric overoxidation or over-reduction. According to the ESCR model [18], this actuating charge density balance during the linear actuation allows a quantitative order for the three studied electrolytes [45] to be established in tendency order LiTFSI (27 mC cm^−2^) > LiTF (23.3 mC cm^−2^) > NaClO_4_ (20.8 mC cm^−2^).

**Figure 4:**
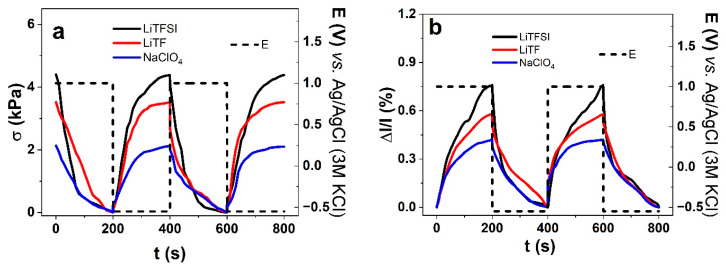

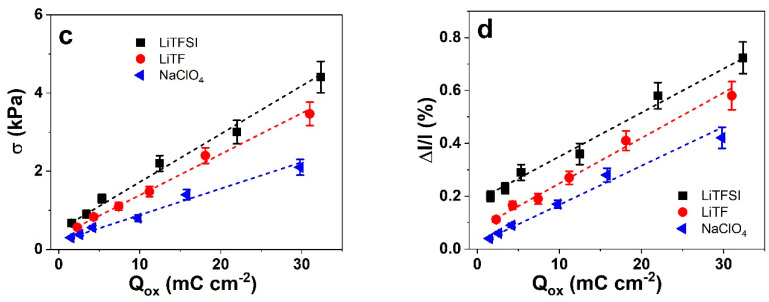
(**a**) Consecutive square potential steps (3rd to 4th) applied to NFs-PPy actuators in three different electrolytes LiTFSI, LiTF, and NaClO_4_ solutions in PC, the applied potential range being 1.0 V to −0.55 V at 2.5 mHz frequency showing stress σ curves and in (**b**) strain Δl/l evolution. The lowest stress points were normalized to zero. The experiments were repeated at different frequencies between 0.0025 Hz and 0.1 Hz. (**c**) Stress gradient evolution as a function of the reaction charge density, Q_ox_, obtained by integration of the chronoamperometric responses (Figure S2c) and (**d**) strain gradient as a function of the charge density. The dashed lines in c and d represent the linear fit.

Figure 4a–d depicts the influence of the applied square potential frequency (0.0025–0.1 Hz). The stress evolution of the NFs-PPy nanofiber at 2.5 mHz is shown in Figure 4a, and the strain evolution in Figure 4b. The stress gradient response to the different applied frequencies is presented in Figure S2a, and those for strain gradients in Figure S2b. From every stationary chronoamperometric response (after applying three consecutive square current waves) to each of the different applied frequencies, the charge density consumed during the PPy oxidation was obtained by integrating the anodic current density/time curves (Figure S2c). As for any other electrochemical actuator or artificial muscle, both stress (Figure 4c) and strain (Figure 4d) gradients are linear functions of the charge density consumed by the actuating electrochemical reaction (oxidation here). The results are presented as mean values with standard deviations from three NFs-PPy independent experimental results in each studied electrolyte.

Figure 4a,b corroborate the results described by voltammetric results. The NFs-PPy actuator expands with oxidation and contracts by electrochemical reduction, indicating a prevalent anion-driven linear actuation. The actuation of the NFs-PPy at 2.5 mHz in the studied electrolytes can be classified as: LiTFSI (stress 4.4 ± 0.40 kPa, strain 0.72 ± 0.06%) > LiTF (stress 3.46 ± 0.32 kPa, strain 0.58 ± 0.05%) > NaClO_4_ (stress 2.12 ± 0.11 kPa, strain 0.42 ± 0.04%). As expected at rising frequencies, the linear actuation presents decreasing stress and strain amplitudes (Figure S2a,b). At low frequencies, oxidation and reduction potentials from each square potential wave are applied longer, consuming higher oxidation and reduction charge densities. This leads to more charged PPy^n+^ chains compensated by a higher number of exchanged anions, giving greater volume increments. From the ESCR model [46], the PPy oxidation–relaxation is promoted as material swelling or shrinking as the higher charge density consumed by the driving reactions at lower frequencies [47]. The NFs-PPy is a faradaic actuator [48] with the charge density consumed by the reactions determining the linear actuation response, Figure 4c,d. The oxidation charge density in LiTFSI solutions at 2.5 mHz was 32.4 ± 2.8 mC cm^−2^, a little higher than that consumed in LiTF solutions with 31.1 ± 2.7 mC cm^−2^ and the lowest found in NaClO_4_ solutions of 29.8 ± 2.4 mC cm^−2^. When electrogenerated, the PPy/DBS gives cation-driven actuation in aqueous electrolytes and prevalent anion-driven actuation in organic (PC) electrolytes [49]. Further investigations were conducted by applying chronopotentiometric measurements.

### 3.3. Chronopotentiometric Measurements of NFs-PPy

The NFs-PPy is a faradaic linear actuator whose consumed charge density should determine the linear actuation. The actuator was submitted to consecutive square current waves using different current densities of ±0.2 A g^−1^ to ±4.0 A g^−1^, consuming a constant charge density of ±40 C g^−1^ every time. From the chronopotentiometry responses in the three studied electrolytes, the NFs-PPy were applied to characterize the dual and simultaneous actuating/sensing properties while storing/releasing electrical energy.

#### 3.3.1. Energy Storage

**Figure 5:**
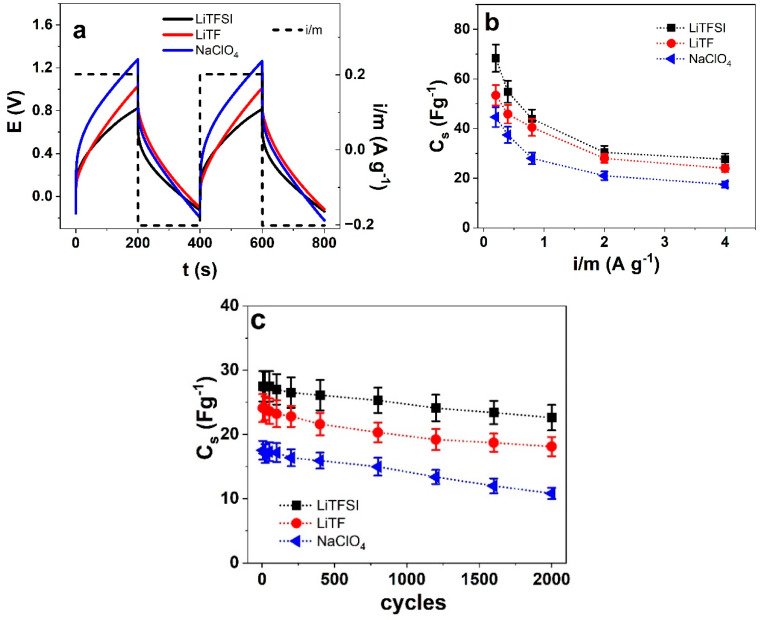
(**a**) Chronopotentiometric responses from NFs-PPy actuators in the three different electrolytes showing two subsequent cycles (3rd and 4th) of the potential time curves at density (i/m) of ±0.2 A g^−1^ (same charge density of 40 C g^−1^). (**b**) The specific capacitance C_s_ is obtained from Equation (2) as a function of the applied current densities i/m. (**c**) Long-term stability of the specific capacitance after applying 2000 cycles (±4.0 A g^−1^) are presented.

The chronopotentiometric (potential/time) responses of the actuating NFs-PPy from the three studied electrolytes when submitted to consecutive square current waves of ±0.2 A g^−1^ are depicted in Figure 5a. From the response to the cathodic current (polymeric reduction or discharging curve), the slope (after IR drop) gives the specific capacitance C_s_ [50] (Equation (2)).

(2) $$C_{s}=\frac{i}{-slope\cdot m}$$

Figure 5b presents the evolution of the specific capacitance as a function of the applied current density. Figure 5c presents the stability of the specific capacitance after 2000 cycles (long-term stability) at ±4.0 A g^−1^.

Figure 5a presents the stationary potential/times (chronopotentiometric) responses related to the third and fourth consecutive square current waves applied to the NFs-PPy in each studied electrolyte using the same oxidation/reduction charge [44]. Figure 5b shows the evolution of specific capacitance in each electrolyte as a function of the applied charge density. The observed fast decrease at rising applied specific currents seems related to the attained high anodic and cathodic potentials where parallel irreversible reactions of the electrolyte components can occur. The specific capacitances attained at ±0.2 A g^−1^ were 68.3 ± 5.5 F g^−1^ in LiTFSI, 53.4 ± 4.1 F g^−1^ in LiTF, and 44.6 ± 3.9 F g^−1^ in NaClO_4_ solutions. This evolution opposes the attained voltages in Figure 5a, supporting that higher voltages originate parallel irreversible reactions that consume charges not used to oxidize and reduce PPy, which stores electrical energy. Previous studies using chemically oxidized PPy on carbon paper gave a similar capacitance [51] of 60 F g^−1^. The specific capacitance of 37.6 F g^−1^ was described from chemically polymerized PPy on activated carbon studied in organic electrolytes [52]. PPy electrochemically polymerized in the presence of polyoxometalates (PTA, phosphotungstic acid), also electroactive, attained up to 80 F g^−1^ (±0.22 A g^−1^) [53]. For PPy/DBS electropolymerized on carbon fiber [54] and studied in aqueous gel electrolytes, a specific capacitance of up to 500 F g^−1^ was attained.

Figure 5c presents the evolution of the NFs-PPy specific capacitance along 2000 charge/discharge cycles at ±4.0 A g^−1^ with 82 % of the capacitance kept for LiTFSI solutions (22.6 ± 2.0 F g^−1^ versus 27.5 ± 2.4 F g^−1^ at the beginning). The 75% capacitance retained in NaClO_4_ (18.1 ± 1.2 F g^−1^ versus 24.1 ± 2.2 F g^−1^) and 61% in LiTF (10.8 ± 0.9 F g^−1^ versus 17.5 ± 1.5 F g^−1^). The specific capacity retention of pseudo-capacitors as chemically formed PPy/graphite in dual ion batteries was 87% (20 A g^−1^) after 3000 cycles in organic electrolytes [55]. PPy chemically generated on twisted cotton fabrics retains 90% of capacity after 2000 charge/discharge cycles at ± 4.0 A g^−1^ cycles in PVA/LiCl solid electrolyte [56]. Those results indicate that our chemically coated PPy on NFs shows positive expectancies for energy storage applications.

#### 3.3.2. Electrical Sensor Calibrations in Different Electrolytes

Chronopotentiometric responses using the same charge/discharge densities allow the simultaneous characterization of sensing-actuating functionalities [19,57] from conducting polymers and other electroactive materials. As happens in natural muscles from any artificial electrochemical actuator (artificial muscles), the driving reaction simultaneously senses the working physical and chemical conditions. The applied current density and the consumed charge density control the actuation rate, and the evolution of the material potential (in fact, that of the reaction energy) responds to and senses the working conditions. Actuating (current) and sensing (potential) signals are present at any actuation time in the two connecting wires, mimicking the muscle’s natural system. In this way, PPy artificial muscles sense trailed weights [58], working temperature [59], and electrolyte concentration [48].

**Figure 6:**
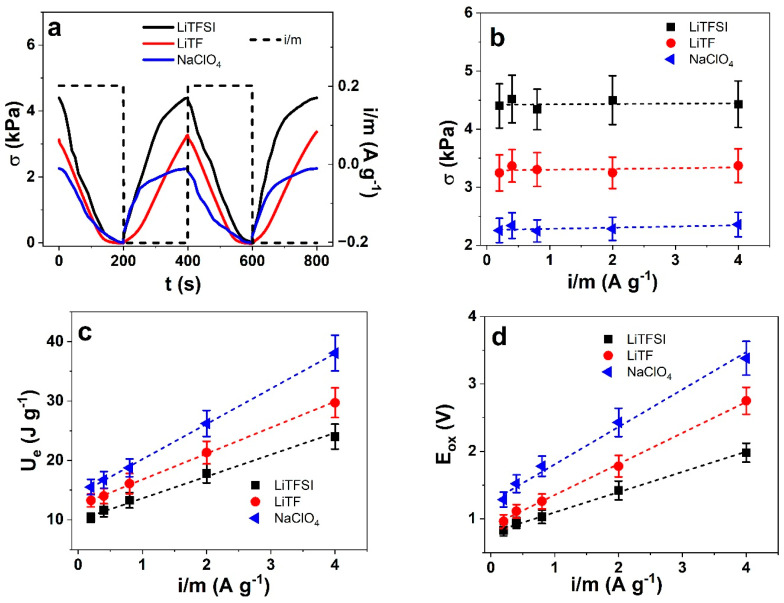
(**a**) Chronopotentiometric responses from NFs-PPy actuators using three different electrolytes LiTFSI, LiTF and NaClO_4_ in PC solvent showing isometric ECMD measurements of stress σ against time (two subsequent cycles 3rd and 4th) by application of consecutive square current density i/m waves of ±0.2 A g^−1^ (dashed black line). (**b**) Average stress gradient is presented as a function of the current density (±0.2 A g^−1^ to ±4.0 A g^−1^) always consuming same charge density of ±40 C g^−1^. Evolution of the consumed reaction electrical energy U_e_ against the current density and d) evolution of the oxidation potential E_ox_ at the end of every anodic chronopotentiogram. The correlation coefficients, R^2^, of the linear fits (dashed lines) range from 0.98 to 0.99 in (**c**,**d**).

Here, the sensing abilities of the NFs-PPy actuators were investigated in the three studied electrolytes consuming the same charge density of ±40 C g^−1^. Figure 6a presents the stress/time responses under the application of ±0.2 A g^−1^. Figure 6b shows the stress gradient as a function of the applied specific currents ranging from ±0.2 A g^−1^ to ±4.0 A g^−1^. The strain/time curves are shown in Figure S3a,b presents the strains attained for different applied current densities. By integrating the potential/time experimental responses to each applied current density, the electrical energy, U_e_, consumed by the actuator in every electrolyte was obtained according to Equation (3).

(3) $$U_{e}\left(t\right)=\frac{i}{m}\int E\left(t\right)dt$$

Figure 6c shows the linear relationships between the actuating reaction energy and the applied current. The material potential at any constant reaction time is a component of the energy the actuating reaction consumes under constant current (Equation (3)). Figure 6d presents the material potential against the applied current densities in the three studied electrolytes.

The stress in Figure 6a and strain in Figure S3a are anion-driven linear actuators. The stress and strain values of NFs-PPy of the three electrolytes in PC can be differentiated. The NFs-PPy are faradaic actuators, and if having the same charge densities (±40 C g^−1^), the linear actuation of either stress (Figure 6b) or strain (Figure S3b) at varied current densities i/m should have a similar response. It is also shown in Figure 6b, regarding stress, and Figure S3b, showing strain, which can be differentiated by the electrolyte applied. From Figure 6b and Figure S3b, the linear adjustment gives the sensor calibration, presented in stress and strain gradients by Table 1. The actuator senses whatever the electrolyte, the working electrical (current here) conditions (Figure 6c) and the potential evolution at oxidation (Figure 6d). The slope of the sensing linear equation is a characteristic of each electrolyte (or exchanged anion).

In short, whatever electrochemical technology is used, the NFs-PPy acts as an anion-driven actuator in the three studied electrolytes, and the magnitude of actuation follows the order TFSI^−^ > TF^−^ > ClO_4_^−^. In the absence of parallel irreversible reactions, the charge consumed by the driving PPy reaction determines the actuation magnitudes [60] that define a faradaic actuator. Both reaction energy and potential evolution adapt to and sense (are linear functions) the working electrical conditions (the current here) to obtain the sensing equations.

For PPy film bending actuators, reaction energy, and potential sense any attached and trailed weight [13]. The differences in the potential time curves in Figure 5a show already different profiles in which electrolyte is applied. Generally, evolutions at higher potential values (ergo high electrical energy consumption), as observed for NFs-PPy in NaClO_4_ solutions, indicate a higher reaction resistivity. Selective anion sensing in organic solvents will open new applications for smart membranes in organic synthesis [61]. The NFs-PPy as an anion-selective sensor can be used in situ during organic reactions or for the anion influence in the hydrophobic core of lipid bilayers in cells [62]. NFs-PPy only requires low voltage for multifunctional approaches such as actuators, sensors, or energy storage for envisaged smart patches in wound-healing monitoring [63].

Nanofibers (NFs) from gelatin type A of porcine skin with glucose are ideal candidates for tissue engineering [23]. However, applications in tissue engineering require biocompatibility and biodegradability, which are compromised by the presence of PPy. Therefore, NFs-PPy composites designed for actuation should focus on bending vibrations, making them suitable as active wound-healing patches (for actuation) and wound monitoring (for sensing) while offering integrated energy storage capabilities. Developing such novel designs is a key direction for future research on NFs-PPy composite materials. Another promising avenue is smart textiles, where these NFs-PPy composites could be woven into fabrics to create healthcare vests capable of sensing heartbeats and monitoring lactose (sweat) concentrations, thereby providing continuous health condition tracking.

## 4. Conclusions

Nanofibers of gelatin type A from porcine skin and glucose (10/1 ratio) were obtained by electrospinning and then coated with polypyrrole (PPy) by chemical oxidation dip-coating. After four consecutive dip-coating steps, the homogenous, smooth appearance of the synthesized PPy attains an average thickness of 0.55 ± 0.04 µm. The isometric and isotonic ECMD characterization of the NFs-PPy state the faradaic nature of the linear actuator and the prevalent anion-driven actuation in PC solutions of three different salts. Stress and strain variations and the charge densities attained simultaneously from cyclic voltammetric and chronoamperometric responses in the studied electrolytes follow LiTFSI > LiTF > NaClO_4_. This sequence is mainly ascribed to the diameter of the anion exchanged by the actuating PPy reaction. During reversible actuation (elongation/contraction), the actuator stores/releases electrical energy, attaining the higher average specific capacitance from chronopotentiometric responses in LiTFSI solutions: 68.3 ± 5.5 F g^−1^ with 82% of capacitance retention after 2000 charge/discharge cycles at ±4.0 A g^−1^. The energy of the actuating reaction or the potential evolution responds to and senses the working electrical conditions. The linear sensing equations were obtained in the three studied electrolytes, and the slopes (sensitivity) were different for each anion. Therefore, the electro-chemo-mechanical characterization of NFs-PPy in solutions of three different salts in PC reveals the simultaneous multifunctionality of the PPy-coated nanofiber as an actuator for energy storage (specific capacitance) and as a sensor. Possible applications are envisaged for health applications, such as smart patches and smart textile applications where nanofibers can be inwoven to provide multifunctionality.

## Figures and Tables

**Table 1:** Sensing equations (linear fit to Equation (3) from Figure 6c,d) of the reaction energy U_e_ consumed by NFs-PPy actuation under oxidation by applying different anodic currents in the three different studied electrolytes (LiTFSI, LiTF and NaClO_4_) solutions in PC). The material potential evolution after 40 C g^−1^ underflow of the different currents is consumed. Average stress σ and strain Δl/l gradients are also presented.

NFs-PPy Electrolytes	U_e_ (J g^−1^)	E_ox_ (V)	Stress σ (kPa)	Strain Δl/l (%)
LiTFSI	$9.93+3.68\frac{i}{m}\left({\mathrm{Ag}}^{-1}\right)$	$0.79+0.30\frac{i}{m}\left({\mathrm{Ag}}^{-1}\right)$	4.42 ± 0.38	0.68 ± 0.06
LiTF	$12.39+4.36\frac{i}{m}\left({\mathrm{Ag}}^{-1}\right)$	$0.89+0.46\frac{i}{m}\left({\mathrm{Ag}}^{-1}\right)$	3.29 ± 0.31	0.49 ± 0.04
NaClO_4_	$14.25+5.94\frac{i}{m}\left({\mathrm{Ag}}^{-1}\right)$	$1.25+0.55\frac{i}{m}\left({\mathrm{Ag}}^{-1}\right)$	2.27 ± 0.21	0.28 ± 0.02

## Data Availability

The original contributions presented in this study are included in the article/Supplementary Material. Further inquiries can be directed to the corresponding author.

## Supplementary Materials

The following supporting information can be downloaded at: https://www.mdpi.com/article/10.3390/polym17050631/s1, Figure S1: SEM image (scale bar 4 µm) of NFs with average thickness of fiber at the range of 0.8 ± 0.06 µm; Figure S2: Square wave potential steps with isotonic ECMD measurements of NFs-PPy at applied potential E (1.0 V to −0.55 V) in three different electrolytes LiTFSI (··■··), LiTF (··●··) and NaClO_4_ (··◄··) using the solvent PC, showing the evolution of stress σ in (a) and the strain ε in (b) against applied frequencies (0.0025 Hz to 0.1 Hz) and the current density j against time at frequency 0.0025 Hz at applied potential E (dashed black line) in (c); Figure S3: Chronopotentiometric measurements with included isotonic ECMD measurements of NFs-PPy at different electrolytes LiTFSI (black line, ■), LiTF (red line, ●) and NaClO_4_ (blue line, ◄) in PC solvent showing the strain Δl/l against time (two subsequent cycles 3rd and 4th) at applied current density i/m (dashed black line) of ± 0.2 A g^−1^ in (a). At constant charge density ± 40 C g^−1^ the strain Δl/l against the current densities i/m (± 0.2 A g^−1^ to ± 4.0 A g^−1^) is presented in (b). The dashed lines in b represent the linear fit.

## References

[1] Melling D., Martinez J.G., Jager E.W.H. (2019). Conjugated Polymer Actuators and Devices: Progress and Opportunities. Adv. Mater..

[2] Jager E.W.H., Inganas O., Lundstrom I. (2000). Microrobots for Micrometer-Size Objects in Aqueous Media: Potential Tools for Single-Cell Manipulation. Science (80-).

[3] García-Córdova F., Guerrero-González A., Zueco J., Cabrera-Lozoya A. (2023). Simultaneous Sensing and Actuating Capabilities of a Triple-Layer Biomimetic Muscle for Soft Robotics. Sensors.

[4] Wang Y., Liu A., Han Y., Li T. (2020). Sensors Based on Conductive Polymers and Their Composites: A Review. Polym. Int..

[5] Shi Y., Peng L., Ding Y., Zhao Y., Yu G. (2015). Nanostructured Conductive Polymers for Advanced Energy Storage. Chem. Soc. Rev..

[6] Kiefer R., Harjo M., Otero T.F., Nguyen N.T., Le Q.B. (2024). Linear Sensing Actuators of Polypyrrole Nanofiber Scaffolds. J. Appl. Polym. Sci..

[7] Hara S., Zama T., Takashima W., Kaneto K. (2004). Artificial Muscles Based on Polypyrrole Actuators with Large Strain and Stress Induced Electrically. Polym. J..

[8] Zama T., Tanaka N., Takashima W., Kaneto K. (2006). Fast and Large Stretching Bis(trifluoromethylsulfonyl)imide (TFSI)-Doped Polypyrrole Actuators and Their Applications to Small Devices. Polym. J..

[9] Hara S., Zama T., Takashima W., Kaneto K. (2005). Free-Standing Polypyrrole Actuators with Response Rate of 10.8% S^−1^. Synth. Met..

[10] Sadki S., Schottland P., Brodie N., Sabouraud G. (2000). The Mechanisms of Pyrrole Electropolymerization. Chem. Soc. Rev..

[11] Heinze J., Frontana-Uribe B.A., Ludwigs S. (2010). Electrochemistry of Conducting PolymerssPersistent Models and New Concepts. Chem. Rev..

[12] Yoon C.O., Sung H.K., Kim J.H., Barsoukov E., Kim J.H., Lee H. (1999). The Effect of Low-Temperature Conditions on the Electrochemical Polymerization of Polypyrrole Films with High Density, High Electrical Conductivity and High Stability. Synth. Met..

[13] Harjo M., Zondaka Z., Leemets K., Järvekülg M., Tamm T., Kiefer R. (2020). Polypyrrole-Coated Fiber-Scaffolds: Concurrent Linear Actuation and Sensing. J. Appl. Polym. Sci..

[14] Stejskal J., Trchová M. (2018). Conducting Polypyrrole Nanotubes: A Review.

[15] Hsu C.F., Zhang L., Peng H., Travas-Sejdic J., Kilmartin P.A. (2008). Scavenging of DPPH Free Radicals by Polypyrrole Powders of Varying Levels of Overoxidation And/or Reduction. Synth. Met..

[16] Trung V.Q., Hung H.M., Van Khoe L., Duc L.M., Bich Viet N.T., Linh D.K., Huong V.T., Dat N.D., Yen Oanh D.T., Luong N.X. (2022). Synthesis and Characterization of Polypyrrole Film Doped with Both Molybdate and Salicylate and Its Application in the Corrosion Protection for Low Carbon Steel. ACS Omega.

[17] Bay L., Jacobsen T., Skaarup S., West K. (2001). Mechanism of Actuation in Conducting Polymers: Osmotic Expansion. J. Phys. Chem. B.

[18] Otero T.F., Boyano I. (2003). Comparative Study of Conducting Polymers by the ESCR Model. J. Phys. Chem. B.

[19] Otero T.F., Martinez J.G. (2015). Physical and Chemical Awareness from Sensing Polymeric Artificial Muscles. Experiments and Modeling. Prog. Polym. Sci..

[20] Misir M., Aydogdu N., Demir E., Manjunatha J.G. (2024). Polymer Functionalized Materials for Design of Electrochemical Sensing Devices. Real-Time Applications of Advanced Electrochemical Sensing Devices.

[21] Siimon K., Reemann P., Pōder A., Pook M., Kangur T., Kingo K., Jaks V., Möeorg U., Järvekülg M. (2014). Effect of Glucose Content on Thermally Cross-Linked Fibrous Gelatin Scaffolds for Tissue Engineering. Mater. Sci. Eng. C.

[22] Harjo M., Tamm T., Anbarjafari G., Kiefer R. (2019). Hardware and Software Development for Isotonic Strain and Isometric Stress Measurements of Linear Ionic Actuators. Polymers.

[23] Dehghanpour H., Yilmaz K. (2020). The Relationship between Resistances Measured by Two-Probe, Wenner Probe and C1760-12 ASTM Methods in Electrically Conductive Concretes. SN Appl. Sci..

[24] Temmer R., Must I., Kaasik F., Aabloo A., Tamm T. (2012). Combined Chemical and Electrochemical Synthesis Methods for Metal-Free Polypyrrole Actuators. Sens. Actuators B Chem..

[25] Harjo M., Kesküla A., Leemets K., Khorram M.S., Saar R., Järvekülg M., Tamm T., Kiefer R. (2017). Polypyrrole Coatings on Gelatin Fiber Scaffolds: Material and Electrochemical Characterizations in Organic and Aqueous Electrolyte. Synth. Met..

[26] Lee J.Y., Bashur C.A., Goldstein A.S., Schmidt C.E. (2009). Polypyrrole-Coated Electrospun PLGA Nanofibers for Neural Tissue Applications. Biomaterials.

[27] Shafei S., Foroughi J., Chen Z., Wong C.S., Naebe M. (2017). Short Oxygen Plasma Treatment Leading to Long-Term Hydrophilicity of Conductive PCL-PPy Nanofiber Scaffolds. Polymers.

[28] Zarei M., Samimi A., Khorram M., Abdi M.M., Golestaneh S.I. (2021). Fabrication and Characterization of Conductive Polypyrrole/chitosan/collagen Electrospun Nanofiber Scaffold for Tissue Engineering Application. Int. J. Biol. Macromol..

[29] Yao Y., Wang H., Wang R., Chai Y. (2019). Novel Cellulose-Gelatin Composite Films Made from Self-Dispersed Microgels: Structure and Properties. Int. J. Biol. Macromol..

[30] Azizi M., Azimzadeh M., Afzali M., Alafzadeh M., Mirhosseini S.H. (2018). Characterization and Optimization of Using Calendula Officinalis Extract in The Fabrication of Polycaprolactone/Gelatin Electrospun Nanofibers for Wound Dressing Applications ARTICLE INFO ABSTRACT. J. Adv. Mater. Process..

[31] Ibrahim M., Alaam M., El-Haes H., Jalbout A.F., De Leon A. (2006). Analysis of the Structure and Vibrational Spectra of Glucose and Fructose. Eclet. Quim..

[32] Siimon K., Siimon H., Järvekülg M. (2015). Mechanical Characterization of Electrospun Gelatin Scaffolds Cross-Linked by Glucose. J. Mater. Sci. Mater. Med..

[33] Gibot P., Goetz V. (2021). Polypyrrole Material for the Electrostatic Discharge Sensitivity Mitigation of Al/SnO_2_ Energetic Composites. J. Appl. Polym. Sci..

[34] Turczyn R., Krukiewicz K., Katunin A., Sroka J., Sul P. (2020). Fabrication and Application of Electrically Conducting Composites for Electromagnetic Interference Shielding of Remotely Piloted Aircraft Systems. Compos. Struct..

[35] Kostić R., Raković D., Stepanyan S.A., Davidova I.E., Gribov L.A. (1995). Vibrational Spectroscopy of Polypyrrole, Theoretical Study. J. Chem. Phys..

[36] Yang C., Liu P. (2009). Core-Shell Attapulgite@polypyrrole Composite with Well-Defined Corn Cob-like Morphology via Self-Assembling and in Situ Oxidative Polymerization. Synth. Met..

[37] Fu Y., Manthiram A. (2012). Core-Shell Structured Sulfur-Polypyrrole Composite Cathodes for Lithium-Sulfur Batteries. RSC Adv..

[38] Morávková Z., Taboubi O., Minisy I.M., Bober P. (2021). The Evolution of the Molecular Structure of Polypyrrole during Chemical Polymerization. Synth. Met..

[39] Kanaan A.F., Pinho A.C., Piedade A.P. (2021). Electroactive Polymers Obtained by Conventional and Non-Conventional Technologies. Polymers.

[40] Ue M., Murakami A., Nakamura S. (2002). A Convenient Method to Estimate Ion Size for Electrolyte Materials Design. J. Electrochem. Soc..

[41] Marque P., Roncali J., Garnier F. (1987). Electrolyte Effect on the Electrochemical Properties of poly(3-Methylthiophene) Thin Films. J. Electroanal. Chem..

[42] Chaban V. (2015). Solvation of the Fluorine Containing Anions and Their Lithium Salts in Propylene Carbonate and Dimethoxyethane. J. Mol. Model..

[43] Li T., Balbuena P.B. (1999). Theoretical Studies of Lithium Perchlorate in Ethylene Carbonate, Propylene Carbonate, and Their Mixtures. J. Electrochem. Soc..

[44] Valero L., Otero T.F., Martinez J.G., Martínez J.G. (2014). Exchanged Cations and Water during Reactions in Polypyrrole Macroions from Artificial Muscles. ChemPhysChem.

[45] Otero T.F., Martinez J.G. (2013). Structural and Biomimetic Chemical Kinetics: Kinetic Magnitudes Include Structural Information. Adv. Funct. Mater..

[46] West B.J., Otero T.F., Shapiro B., Smela E. (2009). Chronoamperometric Study of Conformational Relaxation in PPy(DBS). J. Phys. Chem. B.

[47] Otero T.F. (2016). Coulovoltammetric and Dynamovoltammetric Responses from Conducting Polymers and Bilayer Muscles as Tools to Identify Reaction-Driven Structural Changes. A Review. Electrochim. Acta.

[48] Martinez J.G., Otero T.F., Jager E.W.H. (2014). Effect of the Electrolyte Concentration and Substrate on Conducting Polymer Actuators. Langmuir.

[49] Kiefer R., Martinez J.G., Kesküla A., Anbarjafari G., Aabloo A., Otero T.F. (2016). Polymeric Actuators: Solvents Tune Reaction-Driven Cation to Reaction-Driven Anion Actuation. Sens. Actuators B Chem..

[50] Kaempgen M., Chan C.K., Ma J., Cui Y., Gruner G. (2009). Printable Thin Film Supercapacitors Using Single-Walled Carbon Nanotubes. Nano Lett..

[51] Izadi-Najafabadi A., Tan D.T.H., Madden J.D. (2005). Towards High Power Polypyrrole/carbon Capacitors. Synth. Met..

[52] Kim K.M., Hur J.W., Jung S.-I., Kang A.S. (2004). Electrochemical Characteristics of Activated carbon/Ppy Electrode Combined with P(VdF-Co-HFP)/PVP for EDLC. Electrochim. Acta.

[53] Le Q.B., Zondaka Z., Harjo M., Nguyen N.T., Kiefer R. (2022). Role of Polyoxometalate Contents in Polypyrrole: Linear Actuation and Energy Storage. Materials.

[54] Zhou W., Han G., Xiao Y., Chang Y., Yuan W., Li Y., Liu C., Zhang Y. (2015). Polypyrrole Doped with Dodecyl Benzene Sulfonate Electrodeposited on Carbon Fibers for Flexible Capacitors with High-Performance. Electrochim. Acta.

[55] Sun T., Sun Q.Q., Yu Y., Zhang X.B. (2021). Polypyrrole as an Ultrafast Organic Cathode for Dual-Ion Batteries. eScience.

[56] Lv J., Zhang L., Zhong Y., Sui X., Wang B., Chen Z., Feng X., Xu H., Mao Z. (2019). High-Performance Polypyrrole Coated Knitted Cotton Fabric Electrodes for Wearable Energy Storage. Org. Electron..

[57] Otero T.F., Cortés M.T. (2003). Artificial Muscles with Tactile Sensitivity. Adv. Mater..

[58] Otero T.F., Cortés M.T. (2003). A Sensing Muscle. Sens. Actuators B Chem..

[59] Shabeeba A., Ismail Y.A. (2022). Chitosan/polypyrrole Hybrid Film as Multistep Electrochemical Sensor: Sensing Electrical, Thermal and Chemical Working Ambient. Mater. Res. Bull..

[60] Martinez J.G., Otero T.F. (2014). Structural Electrochemistry. Chronopotentiometric Responses from Rising Compacted Polypyrrole Electrodes: Experiments and Model. RSC Adv..

[61] Beer P.D., Gale P.A. (2001). Anion Recognition and Sensing: The State of the Art and Future Perspectives. Angew. Chem. Int. Ed.

[62] Wu X., Gilchrist A.M., Gale P.A. (2020). Prospects and Challenges in Anion Recognition and Transport. Chem.

[63] Dalisson B., Barralet J. (2019). Bioinorganics and Wound Healing. Adv. Healthc. Mater..

