# Supplementary material for: Chemically Polymerized Polypyrrole on Glucose-Porcine Skin Gelatin Nanofiber as Multifunctional Electrochemical Actuator-Sensor-Capacitor
Source: Polymers (Basel). 2025 Feb 26;17(5):631. doi: 10.3390/polym17050631 (PMC11902419; doi:10.3390/polym17050631)
Supplement: Supplementary file 1 [file polymers-17-00631-s001.zip › polymers-3478812-supplementary.pdf]

Supplementary

Chemically polymerized polypyrrole on glucose-porcine skin gelatin nanofiber as multifunctional electrochemical actuator-sensor-capacitor

Rudolf Kiefer[1,*], Toribio F. Otero[2], Madis Harjo[3] and Quoc Bao Le[1,4]

[1]Conducting polymers in composites and applications Research Group, Faculty of Applied Sciences, Ton Duc Thang University, Ho Chi Minh City, Vietnam

[2]Centre for Electrochemistry and Intelligent Materials (CEMI), Universidad Politécnica de Cartagena, Aulario II, Paseo Alfonso XIII, E-30203 Cartagena, Murcia, Spain

[3]Intelligent Materials and Systems Lab, Institute of Technology, University of Tartu, Nooruse 1, 50411 Tartu, Estonia

[4] National Institute for Materials Advancement, Pittsburg State University, Pittsburg, KS 66762, USA

*Corresponding author. Tel: +84 792696724. E-mail: rudolf.kiefer@tdtu.edu.vn (Rudolf Kiefer)

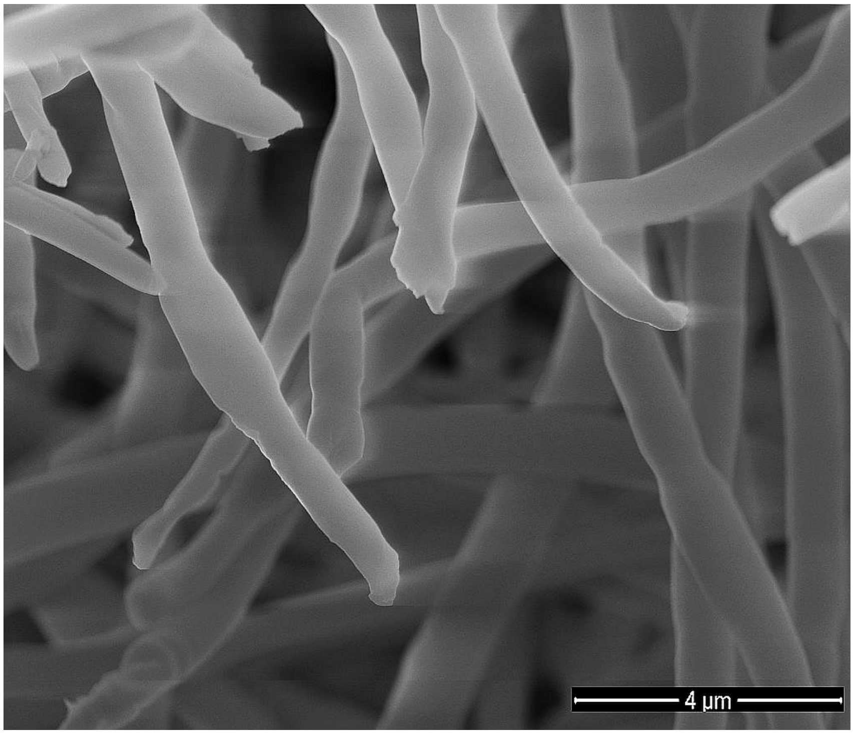

Figure S1. SEM image (scale bar 4 µm) of NFs with average thickness of fiber at the range of 0.8 ± 0.06 µm.

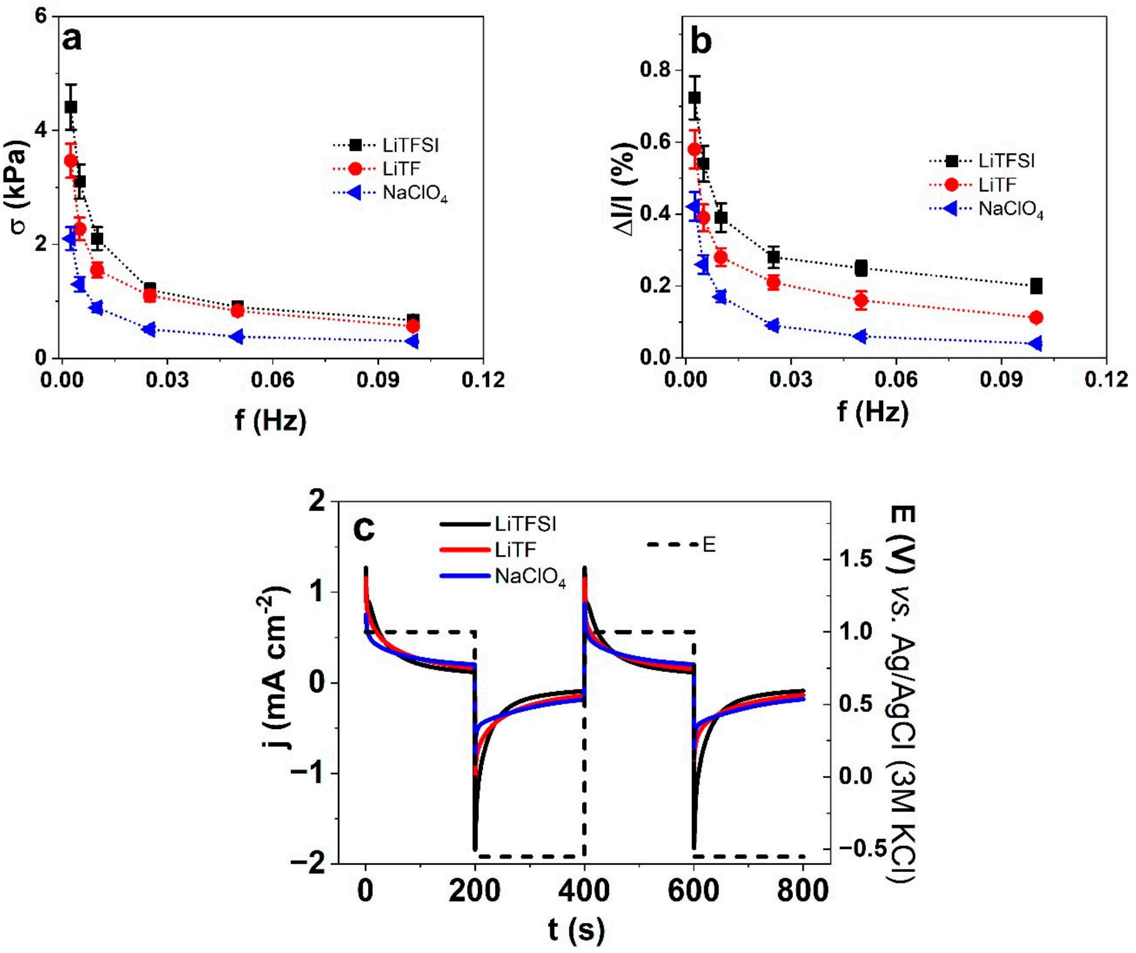

Figure S2. Square wave potential steps with isotonic ECMD measurements of NFs-PPy at applied potential E (1.0 V to -0.55 V) in three different electrolytes LiTFSI (··■··), LiTF (··●··) and $NaClO_4$ (··◀··) using the solvent PC, showing the evolution of stress σ in a) and the strain ε in b) against applied frequencies (0.0025 Hz to 0.1 Hz) and the current density j against time at frequency 0.0025 Hz at applied potential E (dashed black line) in c).

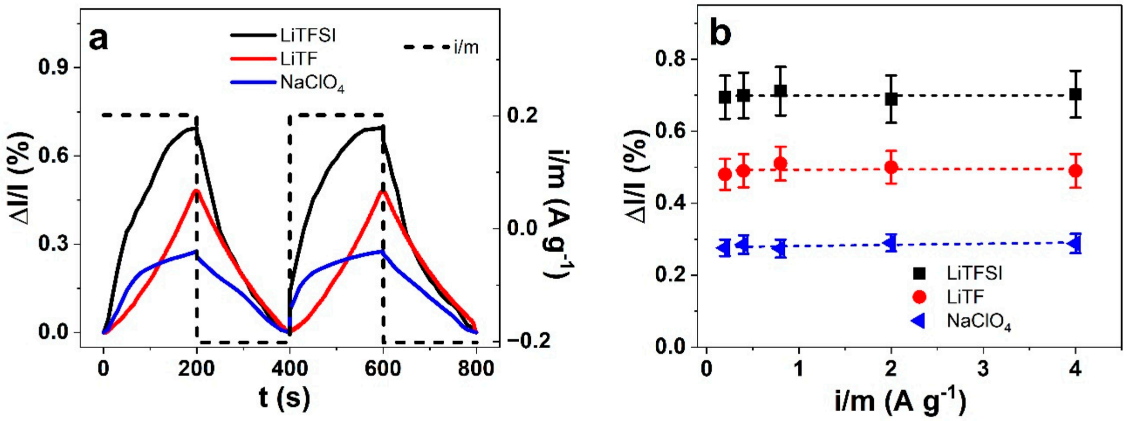

Figure S3. Chronopotentiometric measurements with included isotonic ECMD measurements of NFs-PPy at different electrolytes LiTFSI (black line, ■), LiTF (red line, ●) and $NaClO_4$ (blue line, ◀) in PC solvent showing the strain Δl/l against time (two subsequent cycles 3rd and 4th) at applied current density i/m (dashed black line) of ± 0.2 A g^{-1} in a). At constant charge density ± 40 C g^{-1} the strain Δl/l against the current densities i/m (± 0.2 A g^{-1} to ± 4.0 A g^{-1}) is presented in b). The dashed lines in b represent the linear fit.
